# The Delay of Diagnosis in Spondyloarthropathy Patients in a Tertiary Hospital in Saudi Arabia

**DOI:** 10.7759/cureus.12629

**Published:** 2021-01-11

**Authors:** Mohamed K Bedaiwi, Moath O Baeshen, Amerah Bin Zuair, Reema F AlRasheed

**Affiliations:** 1 Rheumatology Division, Department of Medicine, King Saud University College of Medicine, Riyadh, SAU; 2 Medicine, King Saud University College of Medicine, Riyadh, SAU; 3 Department of Internal Medicine, King Saud University College of Medicine, Riyadh, SAU

**Keywords:** spondyloarthropathy, delay in diagnosis, as, saudi arabia

## Abstract

Objective

Seronegative spondyloarthropathies (SpA) are a group of rheumatological disorders that share the common feature of being rheumatoid factor negative. Inflammation of the sacroiliac joint is considered the hallmark of ankylosing spondylitis (AS). On the other hand, psoriatic arthritis (PsA) affects patients with psoriasis. It is characterized by asymmetrical oligoarticular arthritis. Involvement of the distal interphalangeal joint is a unique feature of PsA. Enteropathic arthritis (EnA) involves the presence of inflammatory arthropathy in patients with inflammatory bowel disease (IBD). These diseases are strongly associated with the HLA-B27 gene. Although they are significantly disabling, their diagnosis has been frequently delayed. Early diagnosis is associated with early treatment, and thus better disease outcomes. The aim of this study was to evaluate the diagnostic delay (DD), that is, the duration between onset of symptoms and diagnosis, of SpA patients and its relation to the demographic characteristics, disease activity, measured by ankylosing spondylitis disease activity score (ASDAS) and bath ankylosing spondylitis disease activity index (BASDAI) scores, and the HLA-B27 status of Saudi SpA patients.

Methods

The data of 94 patients who were diagnosed with SpA were collected from medical records and from them personally. The data included patient demographics, age at diagnosis, delay of diagnosis, in years, disease activity (BASDAI and ASDAS scores), HLA-B27 status and C-reactive protein levels (CRP). The data were analyzed using Statistical Package for the Social Sciences for Windows version 21.0 (SPSS Inc., Chicago, IL, USA).

Results

50% of patients were females. The mean DD was (mean ± SD) 4.98 ± 6.00 (range: 0-35). The average age of symptoms onset was 30.70 ± 11.30 (range: 8-59) and the average age at diagnosis was 35.65 ± 10.80 (range: 16-60). The mean BASDAI and ASDAS scores were 3.05 ± 2.21 and 2.29 ± 1.01, respectively. The majority of the patients had high disease activity (35.1 %). 25.0% were HLA-B27 positive. 83.7 % had normal CRP. There was no statistically significant difference between DD and gender, HLA-B27 status, ASDAS and BASDAI scores, and CRP. The DD was significantly higher in AS patients when compared to PsA (p-value= 0.048) and EnA patients (p-value < 0.0001). There was a statistically significant weak anticorrelation between DD and the age at symptoms onset in PsA patients (r-value= -0.39, p-value= 0.003). Age at diagnosis was statistically significantly higher in patients with PsA when compared to EnA. There was no correlation between DD and the disease activity in SpA patients.

Conclusion

The means of DD in AS, PsA, and EnA patients were 6.69 ± 5.83, 3.67 ± 6.42 and 2.00 ± 1.60, respectively. DD was greater in AS patients when compared to PsA and EnA patients. Early detection and referral to rheumatologists should be addressed, as early intervention is associated with favorable disease outcomes.

## Introduction

Seronegative spondyloarthropathies (SpA) are a group of rheumatological disorders that share some common features. Spondyloarthropathies include ankylosing spondylitis (AS), which is the prototype for SpA, psoriatic arthritis (PsA), reactive arthritis, and enteropathic arthritis (EnA). These diseases affect different joints in the body, with variable frequencies [1]. For instance, they are characterized by inflammatory back pain, due to sacroiliac joint inflammation, asymmetrical oligoarthritis, which majorly affects the lower limb, and enthesitis. In addition, they may involve specific organs resulting in morbidities such as anterior uveitis, psoriasis, and inflammatory bowel disease (IBD). Ankylosing spondylitis (AS) is the most common SpA. In the early stages of the disease, sacroiliitis is considered a hallmark of AS. Genetics appears to have a strong correlation with spondyloarthropathies. The HLA B27, which is an MHC class I molecule, is one of the genetic factors strongly associated with spondyloarthropathies [2].

Diagnostic delay (DD), which is the duration between the onset of the symptoms and the diagnosis, has been influencing different rheumatological diseases. However, AS has been associated with the longest DD, which may result in a greater therapeutic delay [3]. It has been shown that the average delay in AS diagnosis is 8-11 years [2]. This delay has been attributed to different factors, including the delay in seeking medical advice by the patient, the socioeconomic status, and the referral systems are factors that have contributed to the delay in diagnosis [4,5]. The delay in diagnosis has several adverse outcomes. Those with delayed diagnosis showed poor outcomes regarding the activity of their diseases, functional impairment, spinal mobility, and/or radiographic changes. In addition, DD was associated with a poor response to treatment [6]. On the other hand, early diagnosis and treatment may result in better patient outcomes [4].

Different studies have shown variable DD for PsA. In particular, DD values covered a range from several months to about eight years [5,7]. As with AS, DD was related to the delay in seeking medical advice, delay in referral, and the socioeconomic status of the patient [5]. Those who presented two years after the onset of symptoms had more clinical damage and radiographic changes compared to those presenting earlier. This suggests that early diagnosis and onset of treatment will result in better disease outcomes [8].

The number of studies regarding DD in EnA is sparse. Factors that may have a role in DD include the mildness of the initial joint symptoms, the concomitant use of immunosuppressive medications as a treatment for the IBD, and the delay in referral to rheumatologists [9,10]. As with other spondyloarthropathies, shorter disease duration is associated with a better response to treatment and a more favorable disease outcome [10].

To our knowledge, only a few studies have been done to assess DD in patients with SpA in Saudi Arabia, and no previous studies have assessed the differences in DD between AS, PsA and EnA in Saudi Arabia. In this cross-sectional study, we aim to assess the DD of AS, PsA, and EnA patients and its relation to the demographic characteristics, disease activity, measured by ASDAS and BASDAI scores, and HLA-B27 status among Saudi SpA patients.

## Materials and methods

Patients and methods

This was a cross-sectional study on SpA patients following up in the rheumatology clinic of King Khalid University Hospital in Riyadh, Saudi Arabia from February 2018 until February 2019. The total number of patients was 94, 44 of which were diagnosed with AS, 38 with PsA, and 12 with EnA. AS patients who met the Assessment of SpondyloArthritis International Society (ASAS) classification criteria for axial SpA and PsA patients who met the ClASsification criteria for Psoriatic ARthritis (CASPAR) were included in the study [11,12]. Regarding EnA patients, those who were diagnosed with inflammatory bowel disease and presented with joint pain were included in the study. The study was approved by the institutional review board.

Data were collected from the medical records of the patients, direct clinical interviews, and through phone calls. The data collected included the following: patient demographics, smoking status, age at symptoms onset, age at diagnosis, delay of diagnosis, disease activity, HLA-B27 status, and C-reactive protein levels (CRP). The delay of diagnosis is the duration between the age of first having the symptoms and the age of diagnosis in years. The disease activity was assessed by the bath ankylosing spondylitis disease activity index (BASDAI score) and the ankylosing spondylitis disease activity score (ASDAS) [13-15]. ASDAS scores were stratified into: inactive disease (score < 1.3), low disease activity (score 1.3-2.09), high disease activity (score 2.1-3.5) and very high disease activity (score >3.5) [16]. A CRP level greater than 10 mg/L was considered high.

Statistical analysis

All the statistical analyses were performed using the Statistical Package for the Social Sciences for Windows version 21.0 (SPSS Inc., Chicago, IL, USA). A p-value that is lower than 0.05 was considered statistically significant. Data were presented as means and standard deviations for continuous variables, and as frequencies and percentages for categorical and nominal variables. ANOVA was used to compare the 3 spondyloarthropathies with respect to DD. Student t-test was used for continuous variables. χ2 test was used to assess the association between nominal and categorical variables. Pearson-correlation coefficient r was used to assess the linear correlations between paired data-sets.

## Results

Forty-four patients (36.8 %) were diagnosed with AS, 38 (40.4 %) with PsA, and 12 with EnA (12.8 %). Regarding the demographic characteristics, the number of males and females was equal (47 each). About 59.0 % of AS patients were males whilst 63.2 % of the PsA patients were females. The average age of patients was 40.13 ± 11.96 (range: 20-73). Seventeen patients (20.24 %) were smokers or past smokers, of which 13 (76.5 %) were males. The average age at onset of symptoms was [mean ± standard deviation (SD)] 30.70 ± 11.30 (range: 8-59), age of diagnosis was 35.65 ± 10.80 (range: 16-60), DD was 4.87 ± 6.00 (range: 0-35). Sixty-four patients (71.3%) were tested for HLA-B27, and 25.0% were positive. Most of the patients had normal CRP, which was defined as less than 10 mg/L (83.7 %). These results are summarized in Table 1.

**Table 1:** Characteristics of patients with spondyloarthropathies (n = 94). AS: ankylosing spondylitis; PsA: psoriatic arthritis; EnA: enteropathic arthritis; SD: standard deviation; CRP: C-reactive protein; ASDAS: ankylosing spondylitis disease activity score; BASDAI: bath ankylosing spondylitis disease activity index.

	AS		PsA		EnA		Total	
	Mean ± SD or %	Range	Mean ± SD or %	Range	Mean ± SD or %	Range	Mean ± SD or %	Range
Male %	26/44 (59.1 %)		14/38 (36.8%)		7/12 (58.3%)		47/94 (50.0%)	
Age	39.25 ± 10.99	21-63	42.63 ± 12.49	20-73	35.42 ± 12.77	20-58	40.13 ± 11.96	20-73
Age at diagnosis	35.62 ± 9.75	17-75	37.83 ± 11.15	16-60	27.63 ± 11.747	20-55	35.65 ± 10.81	16-60
Delay in Diagnosis (DD)	6.69 ± 5.83	0 - 25	3.67 ± 6.42	0-35	2.00 ± 1.60	0-5	4.98 ± 6.00	0-35
HLA-B27 positive	28.60%		22.70%		14.30%		25.00%	
High CRP	18.20%		11.10%		25.00%		16.30%	
ASDAS	2.17 ± 0.92	0.64-3.99	2.38 ± 1.04	0.15-4.09	2.43 ± 1.28	0.64-4.86	2.29 ± 1.01	0.15-4.86
BASDAI	2.58 ± 1.77	0-6.45	3.43 ± 2.58	0.00-8.85	3.55 ± 2.19	0.6-6.75	3.05 ± 2.21	0.00-8.85
Smoker/past smoker	21.95 %		21.21 %		10.00 %		20.24 %	

A statistically significant relation was absent between DD and gender (p = 0.933). Moreover, there were no significant relationships between HLA-B27 positivity and DD (p = 0.458), age of onset of symptoms (p = 0.301) and age of diagnosis (p = 0.256).

Regarding disease activity, the average BASDAI score was 3.05 ± 2.21, while the average ASDAS score was 2.29 ± 1.01. The means of BASDAI score with their standard deviations were 2.58 ± 1.77, 3.43 ± 2.58, and 3.55 ± 2.19 for AS, PsA, and EnA, respectively. The mean values for ASDAS score were 2.17 ± 0.92, 2.38 ± 1.04, and 2.43 ± 1.28 for AS, PsA, and EnA, respectively. As shown in Table 2, the majority (35.1 %) of the patients had high disease activity. The majority of AS patients had low disease activity (36.1%), while the majority of PsA had high disease activity (40.6 %). Nevertheless, there was no statistically significant correlation between the DD of each of the 3 spondyloarthropathies and both, the BASDAI and ASDAS scores (Table 3). 

**Table 2:** ASDAS groups* * Inactive disease: <1.3; low disease activity: 1.3 - <2.1; high disease activity: 2.1-3.5; very high disease activity: >3.5. ASDAS: ankylosing spondylitis disease activity score.

ASDAS groups	Ankylosing Spondylitis (AS)	Psoriatic Arthritis (PsA)	Enteropathic Arthritis (EnA)	Total
Inactive disease (%)	42.86	42.86	14.29	18.2
Low disease activity (%)	56.52	30.43	13.04	29.9
High disease activity (%)	40.74	48.15	11.11	35.1
Very high disease activity (%)	46.15	46.15	7.69	16.9

**Table 3:** The correlation of diagnostic delay with ASDAS and BASDAI scores among AS, PsA, and EnA patients. DD: delay in diagnosis; AS: ankylosing spondylitis; PsA: psoriatic arthritis; EnA: enteropathic arthritis; ASDAS: ankylosing spondylitis disease activity score; BASDAI: bath ankylosing spondylitis disease activity index.

		AS		PsA		EnA	
		ASDAS	BASDAI	ASDAS	BASDAI	ASDAS	BASDAI
Delay in diagnosis	Pearson correlation	-0.01	0.1	0.07	0.05	-0.11	0.37
	P-value	0.95	0.57	0.73	0.79	0.8	0.37

**Figure 1:**
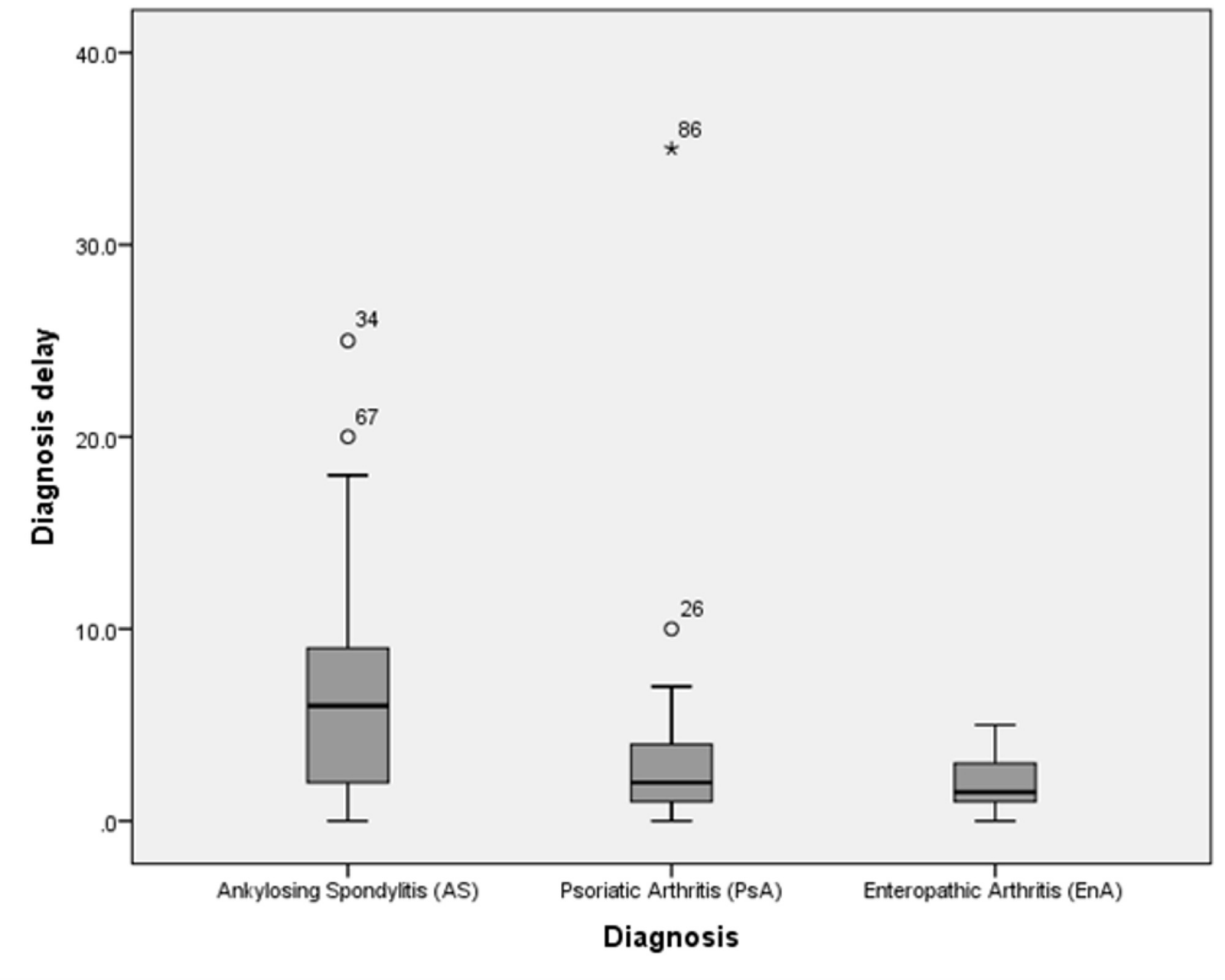
Boxplot comparing the delay in diagnosis in AS, PsA, and EnA patients. AS: ankylosing spondylitis; PsA: psoriatic arthritis; EnA: enteropathic arthritis.

As demonstrated in Table 4, DD was significantly higher in AS patients compared to PsA (p = 0.048) and EnA (p < 0.0001) patients. On the other hand, a statistically significant difference in DD was absent when comparing PsA to EnA (p = 0.475). These results are shown in Figure 1. The age of diagnosis was significantly lower in patients with EnA when compared to AS patients (p = 0.049). Furthermore, a fair anticorrelation was found between DD and age of onset of symptoms in PsA patients (r = - 0.39; p = 0.003) (Table 5).

**Table 4:** The association between age, age at symptoms onset, and age at diagnosis, and delay in diagnosis with AS, PsA, and EnA patients (independent t-test). AS: ankylosing spondylitis; PsA: psoriatic arthritis; EnA: enteropathic arthritis.

	AS and PsA	AS and EnA	EnA and PsA
	P-value	P-value	P-value
Age	0.196	0.305	0.089
Age at symptoms onset	0.058	0.411	0.082
Age at diagnosis	0.39	0.049	0.029
Delay in diagnosis	0.048	0.0001	0.475

**Table 5:** The correlation of diagnostic delay with the age, age at symptoms onset, and age at diagnosis among AS, PsA, and EnA patients. AS: ankylosing spondylitis; PsA: psoriatic arthritis; EnA: enteropathic arthritis.

		AS			PsA			EnA		
		Age	Age at symptoms onset	Age at diagnosis	Age	Age at symptoms onset	Age at diagnosis	Age	Age at symptoms onset	Age at diagnosis
Delay in diagnosis	Pearson correlation	0.12	-0.32	0.27	.42	-0.39	0.15	-0.04	-0.32	-0.2
	p-value	0.48	0.05	0.1	0.03	0.03	0.42	0.92	0.44	0.64

The number of smokers was statistically significantly higher in male patients when compared to females (p = 0.011). However, no statistically significant difference was present between smoking and DD in each of the three diseases (Table 6).

**Table 6:** The association of smoking with the diagnostic delay among AS, PsA, and EnA patients. AS: ankylosing spondylitis; PsA: psoriatic arthritis; EnA: enteropathic arthritis.

		Smoking	Mean	SD	p-value
AS	Delay in diagnosis	No	7.04	5.72	0.77
		Yes	6.31	6.86	
PsA	Delay in diagnosis	No	4.19	7.63	0.61
		Yes	2.4	1.52	
EnA	Delay in diagnosis	No	2	1.79	0.63
		Yes	3	.	

## Discussion

AS

The present study demonstrates a significant DD (6.69 ± 5.83) among Saudi patients with AS. This DD was greater than the delay identified by Omair et al. [17]. In contrast to what was observed by Omair et al., the gender and HLA-B27 were not significantly correlated to factors like age at onset and at diagnosis [17]. Different studies conducted in different countries showed variable delays in the diagnosis of AS. Data from Morocco reported a DD of 4.12 ± 3.99 [4]. Our results were close to those of the Turkish (6.05 ± 5.08), Indian (6.90 ± 5.20), and Japanese (6.70 ± 5.60) populations [18-20]. A longer delay was reported by Seo et al. in Korea, with a mean diagnostic delay of 8 years [6]. In Denmark, DD was reported to be 7.30 ± 6.60 [5]. The RESPONDIA registry, which is an international study involving multiple South American countries, revealed a DD of 8.35 ± 7.85 [7]. Reports from Italy, Germany, and Spain demonstrated longer DD [21-23].

Some studies have shown that HLA-B27 negativity was associated with greater diagnostic delay, nevertheless, the relation was not statistically significant in the present study [18,24]. Similar to the results of Dincer et al. and Ibn Yacoub et al., there was no association between DD and disease activity [4,18]. Although DD was similar to other Asian populations in the present study, it should be interpreted carefully, since our sample size is small.

PsA

Regarding PsA, DD was 3.67 ± 6.42. This was similar to the DD in the Danish population (3.42 ± 4.75) [5]. In addition, another study done in the UK showed a DD of 3.40 years ± 4.10, which is close to both of the aforementioned results [25]. DD of PsA patients was 8.00 ± 7.85 in the RESPONDIA registry [7]. It is important to note that the Danish study and the RESPONDIA registry are population-based studies; hence their results are more representative [5,7]. No statistically significant relation was present between HLA-B27 negativity and DD. However, there appears to be a paucity of the data assessing the association of HLA-B27 and DD in PsA.

EnA

The mean DD of EnA patients was 2.00 ± 1.60. This was lower than that found in RESPONDIA registry (6.05 ± 5.60) [7]. In addition, Conigliaro et al. study revealed a mean DD of 5.20 in the Italian population [26]. Neither HLA-B27 negativity nor disease activity had an impact on DD in our study. Although it is the most common extra-intestinal manifestation of inflammatory bowel diseases, there is scarcity in the data regarding EnA [1].

When the three diseases were compared, DD was significantly higher among AS patients compared to each PsA and EnA patients. A possible explanation is the vagueness of symptoms produced by AS, compared to the cutaneous and gastrointestinal manifestations of PsA and EnA, respectively. It is noteworthy that the DD in each of the three spondyloarthropathies was not prejudiced by gender; however, there is a discrepancy regarding this matter in the literature [5,7,18,19,21,26,27].

One of the major factors contributing to DD is the inability of family physicians and other specialties’ physicians, such as orthopedists, to identify inflammatory back pain and other spondyloarthropathic symptoms [18,19]. Previously, DD did not affect the disease outcome [18,28]. However, today, with the use of anti-TNF agents in the treatment of AS patients, early diagnosis is of great significance, since those with shorter disease duration are more likely to respond to these biological agents [29].

The main strength of our study is that it is the first observational study assessing and comparing the DD among AS, PsA, and EnA patients in Saudi Arabia. Nonetheless, our study was not free of limitations. One of the limitations was recall bias. Patients with longer duration of the disease may have difficulties with remembering their disease onset. However, a study performed on AS patients showed that the majority of them remembered the age at symptoms onset with an accuracy of less than a year [30]. In addition, family history of SpA, which was shown to be associated with a shorter DD, was not taken into account in the study [7,18]. Lastly, since our sample is considered relatively small, the findings of the study should be interpreted with caution.

## Conclusions

The mean duration (years) of DD in AS, PsA, and EnA were 6.69 ± 5.83, 3.67 ± 6.42, and 2.00 ± 1.60, respectively. DD was greater in AS patients when compared to PsA and EnA patients. Early detection and referral to rheumatologists should be addressed, as early intervention favors a better prognosis.
